# Zoledronic acid significantly improves pain scores and quality of life in breast cancer patients with bone metastases: a randomised, crossover study of community *vs* hospital bisphosphonate administration

**DOI:** 10.1038/sj.bjc.6602551

**Published:** 2005-05-03

**Authors:** A Wardley, N Davidson, P Barrett-Lee, A Hong, J Mansi, D Dodwell, R Murphy, T Mason, D Cameron

**Affiliations:** 1 Christie Hospital NHS Trust, 550 Wilmslow Road, Manchester M20 4BX, UK; 2 Broomfield Hospital, Chelmsford CM1 7ET, UK; 3 Velindre Hospital, Cardiff CF14 2TL, UK; 4 Royal Devon & Exeter NHS Foundation Trust, Exeter EX2 5DW, UK; 5 St George's Hospital, London SW17 ORE, UK; 6 Cookridge Hospital, Leeds LS16 6QB, UK; 7 Novartis Pharmaceuticals UK, Camberley GU16 5SG, UK; 8 Western General Hospital, Edinburgh E4H 2XU, UK

**Keywords:** breast cancer, skeletal metastases, bisphosphonates, bone pain, quality of life

## Abstract

Patients with bone metastases from breast cancer often experience substantial skeletal complications – including debilitating bone pain – which negatively affect quality of life. Zoledronic acid (4 mg) has been demonstrated to reduce significantly the risk of skeletal complications in these patients and is administered via a short, 15-min infusion every 3 weeks, allowing the possibility for home administration. This study compared the efficacy and safety of zoledronic acid administered in the community setting *vs* the hospital setting in breast cancer patients with ⩾1 bone metastasis receiving hormonal therapy. After a lead-in phase of three infusions of 4 mg zoledronic acid in the hospital setting, 101 patients were randomized to receive three open-label infusions in the community or hospital setting, followed by three infusions in the opposite venue (a total of nine infusions). The Brief Pain Inventory (BPI) and the European Organisation for Research and Treatment of Cancer Quality of Life Core Questionnaire 30 (EORTC QLQ-C30) were used to assess potential benefits of zoledronic acid therapy. At study end, analysis of the BPI showed significant reductions in worst pain (*P*=0.008) and average pain in the last 7 days (*P*=0.039), and interference with general activity (*P*=0.012). In each case, there were significantly greater improvements in pain scores after treatment in the community setting compared with the hospital crossover setting for worst pain (*P*=0.021), average pain (*P*=0.003), and interference with general activity (*P*=0.001). Overall global health status showed a significant median improvement of 8.3% (*P*=0.013) at study end. Physical, emotional, and social functioning also showed significant overall improvement (*P*=0.013, 0.005, and 0.043, respectively). Furthermore, physical, role, and social functioning showed significantly greater improvements after treatment in the community setting compared with the hospital crossover setting (*P*=0.018, 0.001, and 0.026, respectively). There was no difference between hospital and community administration in renal or other toxicity, with zoledronic acid being well tolerated in both treatment settings. These data confirm the safety and quality-of-life benefits of zoledronic acid in breast cancer patients with bone metastases, particularly when administered in the community setting.

Each year, more than one million women will develop breast cancer worldwide with nearly half of these diagnoses occurring in the United States and Europe. Ultimately, more than 400 000 of these women will die of their disease (Parkin *et al*, 2005). Globally, breast cancer has the highest incidence of all cancers and is the leading cause of cancer mortality in women, accounting for approximately 23% of new cancer cases and approximately 14% of cancer deaths (Parkin *et al*, 1999). Metastasis to bone is common during disease progression and affects an estimated 65–75% of patients with advanced breast cancer (Coleman, 2001). Resulting bone lesions lead to substantial skeletal complications that negatively affect quality of life (Coleman, 2001). Median survival for patients with advanced breast cancer is approximately 18–26 months after the initial diagnosis of bone metastases, placing patients at long-term risk of developing skeletal complications (Domchek *et al*, 2000; Coleman, 2001). Therefore, treatment and prevention of skeletal complications could improve quality-of-life outcomes and result in clinical benefits for these patients.

Zoledronic acid is a new-generation nitrogen-containing bisphosphonate with evidence of significant efficacy in the treatment of bone lesions from multiple myeloma or a variety of solid tumours, including breast, prostate, lung, and renal cell cancer (Lacerna and Hohneker, 2003). In a long-term, randomised, phase III clinical trial, zoledronic acid (4 mg via 15-min infusion) was superior to pamidronate (90 mg via 2-h infusion), the previous standard of care, for reducing the risk of skeletal complications in patients with breast cancer metastatic to bone (Rosen *et al*, 2003, 2004).

Several studies have demonstrated that bisphosphonates have an analgesic effect in patients with bone metastases. In a long-term follow-up of two large, randomised trials in breast cancer patients, pamidronate (90 mg) significantly improved pain scores and reduced need for palliative radiation therapy compared with placebo (*P*<0.001) (Hortobagyi *et al*, 1998; Theriault *et al*, 1999; Lipton *et al*, 2000). In studies comparing zoledronic acid (4 mg) with pamidronate (90 mg) in patients with bone lesions from multiple myeloma or breast cancer, zoledronic acid significantly reduced the need for radiation to bone compared with pamidronate (19 *vs* 24% for pamidronate; *P*=0.037), and was at least as effective as pamidronate for the palliation of bone pain. Brief Pain Inventory (BPI) pain scores improved in 53–69% of patients with pain scores greater than zero at baseline, with no significant differences between treatment groups (Rosen *et al*, 2001, 2003).

In recent years, interest has increased in home care as an alternative to hospital treatment. Several countries, including the United Kingdom, Australia, and the United States, have developed programmes to address this issue. In the United Kingdom, the National Health Service launched a Cancer Plan designed to improve patient access to care by providing home and community treatment options (National Health Service, 2000). Australia launched the Hospital in the Home pilot programme to provide short-term home care as an alternative to hospitalisation (Department of Human Services, 2003). Similarly, the US Medicare system provides short-term Post-Acute Care benefits for homebound individuals (Liu *et al*, 1999). Home care can provide several advantages for patients and caregivers. In several studies, home care was as effective as hospital care and resulted in fewer hospitalisations, decreased pain, improved quality of life, increased performance status, and greater patient satisfaction (Vinciguerra *et al*, 1986; Ventafridda *et al*, 1989; Hughes *et al*, 1992; Shepperd *et al*, 1998; MacIntyre *et al*, 2002).

The 15-min infusion time required for zoledronic acid treatment makes it ideal for home or community administration, particularly for patients not receiving chemotherapy. Therefore, this study investigated the efficacy and safety of zoledronic acid administered in the community setting compared with the hospital setting in breast cancer patients with ⩾1 bone metastasis receiving hormonal therapy.

## PATIENTS AND METHODS

### Patients

The study enrolled adult patients with a histologically confirmed diagnosis of breast cancer and ⩾1 bone metastasis confirmed by conventional radiograph. Patients had to be receiving hormonal therapy for their breast cancer and have an Eastern Cooperative Oncology Group (ECOG) performance status ⩽2. Patients were not eligible if they were receiving chemotherapy or had abnormal renal function, defined as a serum creatinine level >1.5 times the upper limit of normal or a calculated creatinine clearance of <60 ml min^−1^.

### Study design and treatment schedule

**Figure 1:**
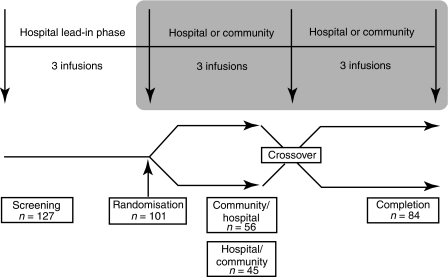
Study design. Zoledronic acid (4 mg) was administered intravenously via 15-min infusion every 3–4 weeks for a maximum of nine infusions. The study was divided into a hospital lead-in phase with three infusions and two community *vs* hospital crossover phases with three infusions in each setting for a total of nine infusions.

This was a phase IIIb, multicentre, randomised, open-label, crossover study. The objective of the study was to determine efficacy and safety of zoledronic acid administered in the community setting *vs* the hospital setting. Patients received zoledronic acid (4 mg) via 15-min intravenous infusion every 3 weeks for up to 9 months. All patients received treatment in the hospital setting for up to three cycles (hospital lead-in phase) to ensure disease stabilisation on hormone therapy. Patients were then randomised to receive treatment for three cycles in either the community setting or the hospital setting. After the three cycles, patients were crossed over to receive three cycles of treatment in the opposite setting. Thus, patients received a total of nine infusions over the course of the study (Figure 1). Infusion of zoledronic acid in the community setting was carried out by nurses from Healthcare at Home Limited (Burton-upon-Trent, UK). This study adhered to Good Clinical Practice (Declaration of Helsinki, Directive 91/507/EEC) and was approved by Multi-Research Ethics Committee. All patients signed informed consent.

### Efficacy assessment

The primary end point was to compare the efficacy of zoledronic acid administered in the community setting with the hospital setting. This end point was measured by evaluating bone pain, quality of life, performance status, resource utilisation, and patient satisfaction. Pain was assessed using the BPI, which measures intensity of pain and interference of pain with daily functioning (Cleeland and Ryan, 1994). Patients rated their pain and the degree to which pain limited their function at the time of response to the questionnaire, as well as their worst, least, and average pain over the previous 7 days. Quality of life was measured using the European Organisation for Research and Treatment of Cancer (Quality of Life Core Questionnaire 30 (EORTC QLQ-C30) and the corresponding disease-specific BR23 breast cancer module (Aaronson *et al*, 1993; Sprangers *et al*, 1996). The EORTC QLQ-C30 questionnaire incorporates nine multi-item scales: five functional scales (physical, role (work and household activities), cognitive, emotional, and social); three symptom scales (fatigue, pain, and nausea and vomiting); and a global health and quality-of-life scale (Aaronson *et al*, 1993). The BR23 breast cancer module consists of 23 items covering symptoms and side effects related to different treatment modalities, body image, sexuality, and future perspective (Sprangers *et al*, 1996). The BPI was assessed at baseline, at the end of each cycle, and at the final visit. The EORTC QLQ-C30 and BR23 questionnaires were assessed at baseline, at the end of each treatment phase (hospital lead-in, community crossover, and hospital crossover), and at the final visit. Analyses of BPI and EORTC QLQ-C30 data were compiled for the 10-visit observation period: three cycles of the hospital lead-in phase, three cycles of the community crossover phase, three cycles of the hospital crossover phase, and final visit. Performance status was assessed using the ECOG scale. Resource utilisation was assessed by calculating time spent travelling to the hospital to receive the three infusions after randomisation.

### Safety assessment

The secondary end point was the safety and tolerability of zoledronic acid and was assessed by monitoring serum creatinine, calculated creatinine clearance, and the occurrence of adverse/serious adverse events. Serum creatinine was measured in the community setting using the i-STAT point-of-care analyser (Abbott Diagnostics, Abbott Park, Il, USA). Decreased renal function was defined as an increase in serum creatinine of ⩾44 or ⩾88 *μ*mol l^−1^ from baseline for patients with baseline serum creatinine of <124 or ⩾124 *μ*mol l^−1^, respectively, or at least twice the baseline value. These criteria were standard across all Novartis zoledronic acid registration protocols. Decreased renal function as measured by calculated creatinine clearance was defined as ⩾1 result more than 30% below baseline. Creatinine clearance was calculated using the Cockcroft-Gault equation. A serious adverse event was defined as any fatal or life-threatening event, any event that required a prolonged hospitalisation, any event that was significantly or permanently disabling or incapacitating, or any event that required medical or surgical intervention to prevent death, disability, or incapacitation.

### Statistical analysis

The efficacy analysis was carried out on an intention-to-treat (ITT) population defined as all patients who received ⩾1 dose of trial medication, provided baseline efficacy data, and from whom ⩾1 postbaseline measurement was obtained. The end point measurement for each randomised patient was the last postrandomisation measurement carried forward to the end of the 3-month phase (hospital or home care). Patients in the ITT population had to provide efficacy data in all phases of the study.

Raw scores from the EORTC QLQ-C30 questionnaires were transformed according to standard methods (Aaronson *et al*, 1993) to produce derived scores (range, 0–100). The derived scores were analysed using a mixed-effects model, in which period (second or third set of three cycles) and treatment (community care or hospital care) were fitted as fixed effects and subject fitted as a random effect. Mean differences between treatments (community care minus hospital care) were calculated and tested by analysis of variance. Model assumptions were checked and found to be adequately satisfied. Analyses were carried out using PROC MIXED of SAS®. For BPI, the composite pain score was derived from the raw scores and analysed using a similar mixed model. All hypothesis tests for the efficacy and safety analyses were two-tailed, *α*=0.05. Novartis UK Medical Information Processing and Statistics carried out statistical analysis using SAS® software, version 8.2 (SAS Institute Inc.).

## RESULTS

### Patients

A total of 127 patients were screened, and 101 patients with ⩾1 bone lesion secondary to breast cancer were enrolled (Figure 1). After the hospital lead-in phase, patients were randomised to treatment with 4 mg zoledronic acid administered in the community setting followed by the hospital setting (*n*=56) or administered in the hospital setting followed by the community setting (*n*=45). A total of 26 patients could not be randomised because of early progression of disease during the three initial hospital infusions. Of the 101 enrolled patients, 84 (83%) completed the study (44 patients in the community/hospital group and 40 patients in the hospital/community group) and 79 (78%) patients were available for analysis, constituting the ITT population. Patient demographics and baseline disease characteristics were similar between treatment groups (Table 1).

### Safety

**Figure 2:**
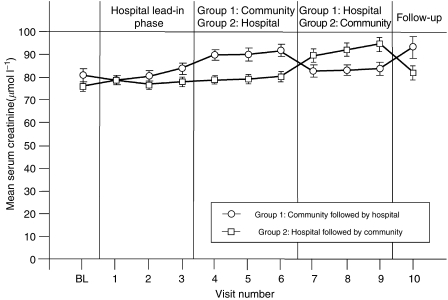
Mean serum creatinine values remained stable throughout treatment with zoledronic acid. Serum creatinine values were measured at baseline (BL), after each cycle, and at follow-up. Measurements during home administration were performed using an i-STAT handheld analyser (Abbott Diagnostics).

Zoledronic acid (4 mg) was well tolerated. Renal function was normal throughout the study for the majority of patients. Serum creatinine levels increased in only four of the 127 (3%) evaluable patients by >44 *μ*mol l^−1^ above baseline. Mean serum creatinine values were higher in the community phase of both treatment groups compared with the hospital lead-in phase or the hospital crossover phase; however, these fluctuations were related to the use of an i-STAT handheld analyser (Abbott Diagnostics). The differences between community and hospital serum creatinine values were not significant and levels returned to near baseline when patients returned to the hospital setting (Figure 2). All mean serum creatinine values were within the generally recognised normal range (54–98 *μ*mol l^−1^). Creatinine clearance was also normal for most patients; only 12 (9.4%) patients had one or more results >30% below baseline, and the majority of these patients only had one such decrease. Adverse events (regardless of relationship to study drug) were mild – most commonly flu-like symptoms, nausea, arthralgia, headache, pain, and vomiting (Table 2). Severe adverse events were experienced by 32 of 127 (25%) evaluable patients; however, these events did not result in any discontinuations from the study and only two events were related to study medication. All serious adverse events were grade 3; no grade 4 serious adverse events were reported. Eight (6.3%) patients died during the study: seven because of disease progression and one because of acute cardiovascular events not thought to be associated with zoledronic acid or their cancer.

### Efficacy

#### BPI scores

**Figure 3:**
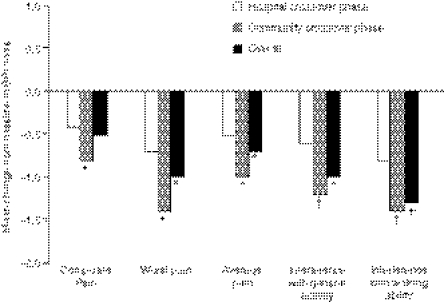
Zoledronic acid significantly improved Brief Pain Inventory (BPI) pain scores. Brief Pain Inventory was assessed at baseline, at the end of each cycle, and at final visit. Graph depicts mean change from baseline BPI scores reported during the hospital crossover phase, community crossover phase, and overall (score reported at final visit after nine infusions). ^*^P<0.05; †*P*<0.005 compared with baseline values.

Over the 10-visit observation period, treatment with zoledronic acid resulted in overall improvement in the composite BPI score in the entire patient population compared with baseline that did not achieve statistical significance (mean, −0.5; *P*=0.077). However, treatment with zoledronic acid resulted in significant reductions in worst pain in the last 7 days (*P*=0.008), average pain in the last 7 days (*P*=0.039), interference with general activity (*P*=0.012), interference with walking ability (*P*<0.001), interference with normal work (*P*=0.005), interference with enjoyment of life (*P*=0.005), and interference with sleep (*P*=0.015) compared with baseline (Figure 3). All other assessments showed small, nonsignificant changes from baseline.

Infusion of zoledronic acid in the community setting achieved significantly greater improvement in BPI pain scores compared with the hospital crossover setting (Figure 3). Significantly greater improvements were reported in the BPI composite score (*P*=0.008), worst pain in the last 7 days (*P*=0.021), average pain in the last 7 days (*P*=0.003), pain right now (*P*=0.013), interference with general activity (*P*<0.001), interference with mood (*P*=0.036), and interference with walking ability (*P*<0.001) in the community crossover phase compared with the hospital crossover phase. In each case, pain scores significantly improved in the community phase compared with baseline, whereas no significant changes were reported in the hospital crossover phase. Interference with normal work improved significantly greater in the community crossover phase compared with the hospital crossover phase (*P*<0.001), and this score improved significantly in both the community crossover phase (*P*=0.011) and the hospital crossover phase (*P*=0.015) compared with baseline.

#### EORTC QLQ-C30 score

**Figure 4:**
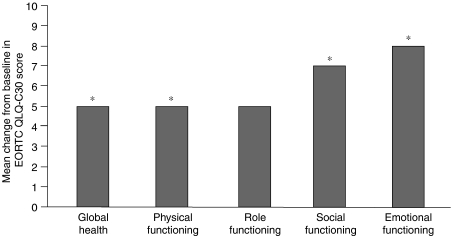
Zoledronic acid significantly improved EORTC QLQ-C30 quality-of-life scores. Graph depicts overall mean change from baseline quality-of-life scores reported at final visit after nine infusions. ^*^P<0.05 compared with baseline values. EORTC QLQ-C30=European Organisation for Research and Treatment of Cancer Quality of Life Core Questionnaire 30.

Over the 10-visit observation period, treatment with zoledronic acid resulted in a significant 5% increase in mean scores for overall global health status compared with baseline (*P*=0.013). Specifically, 36 of the 79 (46%) patients available for analysis reported increases in global health status, 24 (30%) patients reported no change, and 19 (24%) patients reported decreased health status. Assessment of the functional scales showed significant increases in mean scores for physical functioning (6% increase; *P*=0.013), emotional functioning (8% increase; *P*=0.005), and social functioning (7% increase; *P*=0.043) compared with baseline (Figure 4). Overall cognitive and role scales remained stable throughout the course of the study.

**Figure 5:**
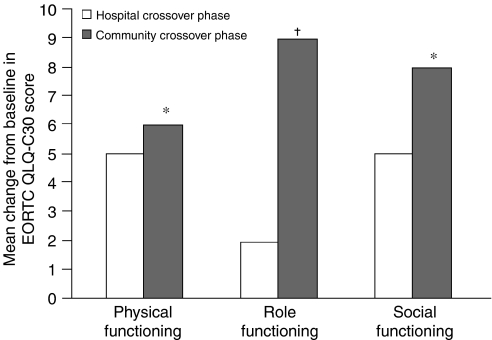
Zoledronic acid achieved significantly greater improvement in EORTC QLQ-C30 quality-of-life scores when administered in the community crossover phase compared with the hospital crossover phase. The EORTC QLQ-C30 questionnaire was assessed at baseline, at the end of each treatment phase (hospital lead-in, community crossover, and hospital crossover), and at final visit. Graph depicts mean change from baseline quality-of-life scores reported during the hospital crossover phase and the community crossover phase. ^*^P<0.05; †*P*<0.001 compared with hospital lead-in score. EORTC QLQ-C30=European Organisation for Research and Treatment of Cancer Quality of Life Core Questionnaire 30.

Infusion of zoledronic acid in the community crossover setting resulted in significantly greater improvement in physical functioning (*P*=0.018), role functioning (*P*=0.001), and social functioning (*P*=0.026) compared with the hospital crossover setting (Figure 5). Physical functioning scores during the community crossover phase showed a significant mean increase of 3% from baseline (*P*=0.002) compared with a nonsignificant improvement in the hospital crossover phase. Similarly, role functioning scores increased significantly *vs* baseline in the community crossover phase (mean increase=8%; *P*=0.007), whereas a nonsignificant increase was reported during the hospital crossover phase. Social functioning scores were stable compared with baseline in both community crossover and hospital crossover phases.

Assessment of patient perception of symptoms revealed significantly greater improvement in pain and diarrhoea scores in the community crossover phase (*P*=0.031) compared with the hospital crossover phase (*P*=0.01). Pain scores improved significantly by a mean of 4% from baseline in the community crossover phase (*P*=0.022) compared with a nonsignificant improvement in the hospital crossover phase. Similarly, diarrhoea scores improved significantly by 5% from baseline in the community phase (*P*=0.005) compared with a nonsignificant improvement during the hospital crossover phase. All other symptoms remained stable, with changes <5%.

Patients also experienced significantly fewer financial difficulties during the community crossover phase of the study compared with the hospital crossover phase (*P*=0.004). During the community crossover phase, patients reported a significant mean decrease of 4% in financial difficulties (*P*=0.029), whereas during the hospital crossover phase a significant mean increase of 5% (*P*=0.003) was reported compared with baseline. For all EORTC QLQ-C30 scales, an increase of >5% was considered to be clinically significant.

#### BR23 breast cancer module

Over the course of the study, patients reported significant declines in future perspective with a median change of 0 and a mean decrease of 17% (*P*<0.0001) in the entire patient population compared with baseline. However, sexual function significantly increased (median change of 0 and mean increase of 4%; *P*=0.049). Body image and systemic therapy side effects remained stable. Hair loss and sexual enjoyment had too few respondents to assess meaningfully. Arm symptoms showed significant differences between the community crossover and hospital crossover settings. In the hospital crossover setting, arm symptoms improved 3% (*P*=0.004), whereas the community setting arm symptoms worsened 4% (*P*=0.076) compared with baseline.

#### ECOG performance status

No significant changes in performance status were noted during the study compared with baseline measurements. The majority of patients were either fully active or restricted in physically strenuous activity.

#### Resource utilisation

Time that patients spent travelling to the hospital was calculated for the three hospital infusions of zoledronic acid after randomisation. Patients spent a cumulative time of more than 240 h travelling to and from the hospital (median, 2.5 h for all three visits; range, 0.25–13.5 h).

#### Patient satisfaction

Overall, patients were satisfied with zoledronic acid treatment (Table 3). Patients were significantly more satisfied when zoledronic acid was administered in the community crossover setting compared with the hospital crossover setting. Significant differences were noted in the percentage of patients >80% satisfied (94% for community *vs* 83% for hospital crossover; *P*=0.048) and 100% satisfied (73% for community crossover *vs* 51% for hospital crossover; *P*=0.005).

## DISCUSSION

Patients with breast cancer are at high risk for bone metastases, resulting in significant skeletal complications and bone pain that negatively affects their quality of life. Long-term treatment with zoledronic acid (4 mg) has been shown to reduce the risk of skeletal complications in these patients by an additional 20% compared with pamidronate, particularly in patients receiving hormonal therapy, in whom the risk was reduced by an additional 30% (Rosen *et al*, 2003). Zoledronic acid is suited to home administration because of its short infusion time and its favourable safety profile. Furthermore, patients with breast cancer receiving hormonal therapy are particularly suited to home care because of the apparent earlier stage of their disease and better prognosis *vs* patients receiving chemotherapy. Therefore, this randomised crossover study investigated the efficacy and safety of zoledronic acid in the community setting *vs* the hospital setting in breast cancer patients with bone metastases receiving hormonal therapy. Results showed that zoledronic acid was safe in both the community and hospital settings, and analysis of BPI and EORTC QLQ-C30 quality-of-life scores demonstrated that zoledronic acid significantly improved composite pain scores and overall quality of life compared with baseline, particularly when administered in the community setting.

In this study, zoledronic acid was safe and well tolerated; adverse events were mild, and no patient experienced a sustained decrease in renal function in either the community or hospital setting. Fluctuations in home serum creatinine measurements were noted but were related to the use of an i-STAT handheld analyser. Importantly, mean serum creatinine values were always within the normal range and returned to baseline during infusions in the hospital, where values were measured in the hospital laboratory.

Analysis of BPI scores showed no changes in the overall composite score at end of study compared with baseline; however, significant decreases were noted in several subcategories. Notably, a significant improvement was observed in many aspects of pain, although these scores were relatively low at baseline. A recent analysis of treatment with zoledronic acid in women with metastatic breast cancer demonstrated that better baseline scores were associated with less improvement after treatment, whereas poorer initial scores were associated with higher rates of change (Weinfurt *et al*, 2004). Therefore, although the improvements in pain scores reported by patients in this trial were small, the differences were significant, demonstrating that the overall well-being of these patients improved. Comparison of community *vs* hospital infusion of zoledronic acid showed that composite pain scores significantly improved in the community setting. This result confirms a previous study demonstrating that home care of patients with advanced cancer decreased narcotic and analgesic requirements compared with hospital care (Vinciguerra *et al*, 1986), and potentially reflects the increased satisfaction often experienced by patients receiving home care (Ventafridda *et al*, 1989; Hughes *et al*, 1992).

Previously, the EORTC QLQ-C30 quality-of-life questionnaire has not been used for the assessment of zoledronic acid efficacy. This questionnaire provides a comprehensive assessment of the quality of life of cancer patients participating in clinical trials. According to this questionnaire, global health status improved significantly over the course of the study, as did physical, social, and emotional functioning (Figure 4). In all cases, these improvements were >5% and considered clinically significant. Furthermore, these improvements were observed despite the fact that quality-of-life scores were only assessed every 3 months, resulting in some variability. These results are consistent with the pivotal phase III study of zoledronic acid *vs* pamidronate in patients with multiple myeloma or breast cancer and bone metastases, wherein significant increases in mean ECOG performance status scores compared with baseline were achieved for both treatment groups between months 15 and 25 (Rosen *et al*, 2003). Notably, physical and role functioning achieved significantly greater improvement with zoledronic acid infusion in the community setting compared with the hospital setting, most likely because community treatment allowed patients to continue with their normal work routine. In a recent randomised, crossover trial of home-based *vs* hospital-based chemotherapy in Australia, home therapy was significantly preferred over hospital therapy (*P*<0.0001) (Rischin *et al*, 2000). Reasons cited for this preference included the elimination of travel, reduced treatment anxiety, reduced caregiver burden, and the ability to continue other duties. The benefit of home care on quality of life has also been documented in other National Health Service programmes developed in the United Kingdom, such as the home dialysis programme for patients with end-stage renal failure instituted by the National Institute for Clinical Excellence (2002).

In addition to improved patient satisfaction and quality of life, home treatment may also reduce patient and healthcare costs. Many studies reported decreased healthcare costs (reductions ranging from 18 to 85%) for treatment provided in the home compared with the hospital (Hughes *et al*, 1992; Ventafridda *et al*, 1989). A randomised trial conducted by the US Department of Veterans Affairs evaluating the cost effectiveness of home care compared with customary hospital care for 171 terminally ill patients reported that patients receiving home care used 5.9 fewer hospital days (*P*=0.03), resulting in a significant 47% per capita savings in hospital costs (*P*=0.02) and an 18% reduction in total per capita healthcare costs (Hughes *et al*, 1992). This study also demonstrated a significant increase in patient (*P*=0.02) and caregiver (*P*=0.005) satisfaction with home care after 1 month of treatment. Therefore, home care may not only result in lowered healthcare costs and reduced expenses, but also a more satisfying and comfortable lifestyle for patients and caregivers.

In summary, zoledronic acid is a safe and effective treatment for skeletal complications resulting from bone metastases in patients with breast cancer. Furthermore, infusion of zoledronic acid in the community setting significantly improved pain and quality of life compared with hospital administration, and patients were more satisfied with treatment in the home. A cost analysis that includes cost of treatment and patient out-of-pocket expenses needs to be conducted to assess fully potential economic benefits for patients and the healthcare system. However, the short infusion time of zoledronic acid and the patient benefits demonstrated by this study suggest that zoledronic acid is an excellent candidate for administration in the community setting.

## Figures and Tables

**Table 1:** Patient demographic and baseline disease characteristics by treatment group

**Characteristic**	**Community/hospital (*n*=56)**	**Hospital/community (*n*=45)**
Mean (s.d.) age (years)	60 (11.8)	59 (10.7)
Range (years)	37–87	37–76
		
*Race, n* (%)		
White	56 (100)	44 (98)
Black	0	1 (2)
		
*ECOG status, n* (%)		
0 or 1	49 (88)	41 (91)
⩾2	6 (11)	4 (9)
Missing but scored 0 at visit 4	1 (1)	0
Mean (s.d.) BPI composite pain score	2.6 (1.7)	2.7 (2.4)
		
*Baseline serum creatinine, n* (%)		
<124 *μ*mol l^−1^	56 (100)	45 (100)

s.d.=standard deviation; ECOG=Eastern Cooperative Oncology Group; BPI=Brief Pain Inventory.

**Table 2:** Adverse events (all grades), regardless of relation to study drug, occurring in ⩾10% of patients (safety-evaluable population)

	**Patients, *n* (%)**
**Adverse event**	**Zoledronic acid 4 mg (*n*=127)**
Any event	121 (95)
Influenza-like illness	40 (31)
Nausea	30 (24)
Arthralgia	27 (21)
Headache	25 (20)
Pain NOS	25 (20)
Vomiting NOS	21 (17)
Back pain	19 (15)
Constipation	17 (13)
Fatigue	17 (13)
Pain in limb	13 (10)

NOS=not otherwise specified.

**Table 3:** Patient satisfaction with zoledronic acid treatment in hospital *vs* community setting

	**Patients, *n* (%)**	
	**Satisfaction >80%**	**Satisfaction=100%**
Hospital lead-in	57 (79)	32 (44)
Community	74 (94)	58 (73)
Hospital crossover	65 (83)	40 (51)
Final visit	72 (91)	51 (65)
